# The value of liver resection for focal nodular hyperplasia: resection yes or no?

**DOI:** 10.1186/s40001-015-0181-x

**Published:** 2015-10-22

**Authors:** Hans Michael Hau, Georgi Atanasov, Hans-Michael Tautenhahn, Rudolf Ascherl, Georg Wiltberger, Markus Bo Schoenberg, Mehmet Haluk Morgül, Dirk Uhlmann, Michael Moche, Jochen Fuchs, Moritz Schmelzle, Michael Bartels

**Affiliations:** Department of Visceral, Transplantation, Vascular and Thoracic Surgery, University Hospital of Leipzig, Liebigstraße 20, 04103 Leipzig, Germany; Translational Centre for Regenerative Medicine, University of Leipzig, Leipzig, Germany; Department of Diagnostic and Interventional Radiology, University Hospital Leipzig, Leipzig, Germany; Department of General, Visceral, Vascular and Thoracic Surgery, Charité-University Hospital of Berlin, Berlin, Germany

**Keywords:** Focal nodular hyperplasia, Liver resection, Quality of life, Diagnostical algorithm

## Abstract

**Background:**

Focal nodular hyperplasia (FNH) are benign lesions in the liver. Although liver resection is generally not indicated in these patients, rare indications for surgical approaches indeed exist. We here report on our single-center experience with patients undergoing liver resection for FNH, focussing on preoperative diagnostic algorithms and quality of life (QoL) after surgery.

**Methods:**

Medical records of 100 consecutive patients undergoing liver resection for FNH between 1992 and 2012 were retrospectively analyzed with regard to diagnostic pathways and indications for surgery. Quality of life (QoL) before and after surgery was evaluated using validated assessment tools. Student’s *t* test, one-way ANOVA, *χ*^2^, and binary logistic regression analyses such as Wilcoxon–Mann–Whitney test were used, as indicated.

**Results:**

A combination of at least two preoperative diagnostic imaging approaches was applied in 99 cases, of which 70 patients were subjected to further imaging or tumor biopsy. In most patients, there was more than one indication for liver resection, including tumor-associated symptoms with abdominal discomfort (*n* = 46, 40.7 %), balance of risk for malignancy/history of cancer (*n* = 54, 47.8 %/*n* = 18; 33.3 %), tumor enlargement/jaundice of vascular and biliary structures (*n* = 13, 11.5 %), such as incidental findings during elective operation (*n* = 1, 0.9 %). Postoperative morbidity was 19 %, with serious complications (>grade 2, Clavien–Dindo classification) being evident in 8 %. Perioperative mortality was 0 %. Liver resection was associated with a significant overall improvement in general health (very good–excellent: preoperatively 47.4 % vs. postoperatively 68.1 %; *p* = 0.015).

**Conclusions:**

Liver resection remains a valuable therapeutic option in the treatment of either symptomatic FNH or if malignancy cannot finally be ruled out. If clinically indicated, liver resection for FNH represents a safe approach and may lead to significant improvements of QoL especially in symptomatic patients.

**Electronic supplementary material:**

The online version of this article (doi:10.1186/s40001-015-0181-x) contains supplementary material, which is available to authorized users.

## Background

Focal nodular hyperplasia (FNH) is the second most common benign lesion of the liver most frequently occurring in healthy, young and middle-aged women [1–3]. As most FNH are asymptomatic and rarely grow, these lesions are often discovered incidentally by routine abdominal ultrasound [3–5]. Although malignancy can safely be ruled out in most cases based on imaging and surveillance of these patients, some cases represent diagnostic challenge even in these days [3–7]. In this context, many different single-center algorithms for the diagnostic and therapeutical management of asymptomatic patients with incidental benign liver tumors have been initiated within the past years, and a consensus paper has recently been published to manage these findings on computed tomography (CT) in asymptomatic patients [4, 8, 9].

Diagnostic tools commonly used over the past years include ultrasound, nuclear medical procedures (hepatobiliary scintigraphy with ^99^Tc) and cross-sectional imaging, e.g. CT. Contrast-enhanced magnetic resonance imaging (MRI) scanning has been shown to be the most sensitive modality for the characterization of FNH and is increasingly applied [10–15]. However, characterization might still be challenging, e.g. for single lesions being too small to characterize (TSTC lesions) or in patients with a history of cancer [13–18].

Furthermore, the differential diagnosis can be difficult in cases of atypical appearance (e.g. missing central scar) or in elderly patients with underlying chronic liver disease at risk for hepatocellular carcinoma (HCC) [16–18]. Invasive diagnostics, e.g. percutaneous biopsy of hypervascular lesions might be associated with bleeding and tumor spread in case of malignancy. Theses techniques may also show unclear or misleading results [2–4, 19–21].

The management of benign liver tumors has changed over time and is still evolving. Considering the indolent natural history of FNH with a low risk for complications and no malignant potential, patients with asymptomatic FNH should indeed be treated conservatively [1–6, 11, 12, 19, 20, 22]. A causal relationship between hormonal contraception and growth of FNH has broadly been discussed controversially by many authors, whereas a minority of physicians might still favor and recommend at first to discontinue hormonal contraceptives [22–24].

Surgical approaches should be considered in case of tumor enlargement (after discontinued hormonal contraception), as these patients are at increased risk for intrahepatic complications, e.g. bile duct compression with resulting cholestasis and for rupture or/and acute bleeding [2–6, 10, 20, 22, 25, 26]. Liver resection might also be indicated if FNH presents with atypical features and malignancy cannot be ruled out [22–26]. Some patients indeed report on severe lesion-associated symptoms, e.g. abdominal pain or obstruction of large vessels or intrahepatic bile ducts and might further benefit from surgical approaches. However, many patients suffer from unspecific abdominal discomforts and it remains uncertain whether these patients really benefit from liver resections [10, 20, 22, 26–29]. Unfortunately, there are only limited data evaluating the benefit-risk balance after liver resections for benign liver lesions [30, 31]. Previous studies investigating QoL after liver resection mostly included patients with malignant tumors [32, 33]. However, to date there are only few studies available investigating QoL improvements after liver resection for FNH [22].

We here report on 100 consecutive patients undergoing liver resection for FNH within the past 20 years at a single center. The retrospective analysis summarizes indications for surgical approaches and the outcome of these patients, especially with regard to their QoL after liver resection. Liver resection for FNH represents a safe approach, being associated with low morbidity and no mortality in our series. Especially symptomatic FNH patients might benefit from liver resection, as shown by significant improvements of their QoL.

## Methods

### Study population

All records of patients undergoing liver resection for histologically proven FNH at the Department of Surgery, University Hospital of Leipzig, between January 1992 and October 2012 were analyzed retrospectively. The study was approved by the local ethical commission board from the University of Leipzig. Patients were subclassified into four different time periods (period 1: 1992–1997, period 2: 1998–2002, period 3: 2003–2007, period 4: 2008–2012), with regard to improved imaging modalities and surgical procedures over the study period.

**Fig. 1:**
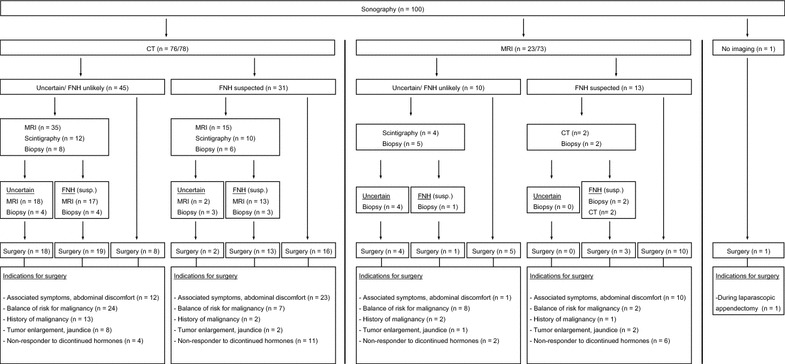
Diagnostic and therapeutical algorithm of our study population

Before liver resection, patients underwent individualized staging procedures, including abdominal ultrasound, CT, MRI, ^99^Tc hepatibiliary scintigraphy and liver biopsy, as indicated (Fig. 1). Symptomatic patients with abdominal discomfort underwent additional endoscopy of the upper and lower GI to exclude extrahepatic disorders. Patients with jaundice were all scheduled for endoscopic retrograde cholangio-pancreatography (ERCP).

### Variables/data collection

Postoperative complications are presented according to the Clavien-Dindo-Classification (grades I–V). The perioperative mortality was defined as 30-day mortality. The following variables were assessed: age (years), body mass index (kg/m^2^), sex (male vs. female), hormonal contraception (yes vs. no), hormonal contraception (years), history of cancer (yes vs. no), preoperative liver blood tests [alanine aminotransferase (ALAT, µmmol/l), aspartat aminotransferase (ASAT, µmmol/l), y-glutamyltransferase (y-GT, µmmol/l), bilirubin (µmmol/l), alkaline phopshatase (AP, µmmol/l), prothrombin time (%)], duration of the operation (min), extent of resection (≥4 segments vs. <4 segments; laparoscopic yes vs. no), red blood cell concentrate (RBCC) intraoperatively (yes vs. no), fresh frozen plasma (FFP) intraoperatively (yes vs. no), tumor size (cm),tumor weight (g), number of lesions (*n*), tumor distribution (unilobular vs. multilobular), length of intensive care unit (ICU) stay (days), length of hospital stay (days) and aminopyrine breath test (ABT).

FNH was verified in all cases after the operation histopathologically. Classical FNH were distinguished from non-classical FNH, whereas non-classical FNH could be divided into three subtypes: teleangiectasic, atypical and mixed (hyperplastic or adenomatous) [1, 2, 34].

### Quality of life assessment

All patients were invited to complete a QoL survey retrospectively (see Additional files 1 and 2). The survey was sent by mail to patients’ home addresses in 2013. Additionally, patients were interviewed via telephone or during clinical visit, as indicated. Patients were asked to evaluate their QoL before and after full recovery from surgery. As no specific questionnaire exists for patients undergoing liver resection for benign liver tumors, a separate questionnaire was designed using elements from the Short-Form 36, McGill Pain Score and European Organization for Research and Treatment of Cancer Quality of Life Questionnaire Core [32, 35, 36]. The survey consists of 44 questions, composed of multi-item scales to evaluate physical, emotional, cognitive and social functioning. The questionnaire also included 5-symptome-scales (bodily pain, nausea/vomiting, abdominal tenderness, fatigue and loss of appetite) as well as global scales of general health concerns, physical health, social elements, body image and overall QoL. Likert scale included items from 0 (none) to 4 (extreme). The survey included questions regarding the pain score before the operation as well as 1, 6 and 12 months after surgery. Pain score involved 5 items ranging from 0 (none) to 4 (extreme).

### Statistical analysis

Statistical data analysis was performed using SPSS software (SPSS Inc., Chicago, Illinois, USA, version 18.0). Mean and median values were used to describe continuous data with discrete variables displayed as total, frequencies and standard deviations (SD)/range where applicable. Student’s *t* test, one-way ANOVA, *χ*^2^ and binary logistic regression such as Wilcoxon–Mann–Whitney test were used where appropriate. Binary logistic regression analysis was performed to test the association of clinical and operative variables with improved QOL, expressed as odds ratios (OR) with a 95 % confidence interval (CI). Two-tailed *p* values <0.05 were considered statistically significant.

## Results

### Demographic and clinicopathological data

According to our retrospective database, 179 patients underwent liver resection for benign lesions between January 1992 and October 2012, of which 100 patients had been diagnosed with FNH. During the investigated time period 243 patients were diagnosed with FNH at our surgical department, of which 41 % (100) patients received an operation. With regard to the different time periods, the number of liver resections for FNH decreased in recent years from 5.9 per year (range 1–38) between 1992 and 2007 to 2.75 per year (range 1–11) between 2008 and 2012 (*p* = *0.19*). Patients’ demographic and clinicopathological data are summarized in Table 1.

**Table 1:** Demographic and clinicopathological characteristics of our study population (*n* = 100 patients)

Variables	Median (range) or *n* (%)
Demographics	
Age (years)	44 (21–71)
Body mass index (kg/m^2^)	24.9 (17.6–35.3)
Gender	
Female	89 (89 %)
Male	11 (11 %)
Laboratory values	
ASAT (µmmol/l)	0.39 (0.2–1.15)
ALAT (µmmol/l)	0.36 (0.15–1.7)
y-glutamyltransferase (µmmol/l)	1.4 (0.1–9.6)
Bilirubin (µmol/l)	8.7 (2–30)
Alkaline phosphatase (µmol/l)	2.5 (0.7–7.1)
Prothrombin time (%)	104 (58–134)
Pathology	
Typical FNH	80 (80 %)
Non-classical FNH	20 (20 %)
Tumor size (cm)	5.9 (2–17)
Tumor distribution	
Unilobular	76 (76 %)
Multilobular	24 (24 %)
Tumor weight (g)	366.5 (10–1886)
Number of tumor lesions	1.59 (1–7)
Medical history	
Previous hormone therapy	
Yes	67 (67 %)
No	33 (33 %)
Median time of hormone use (years)	18 (1–35)
Other medicaments (aspirine, steroids, antidepressants)	14 (14 %)
History of cancer	
Yes	18 (18 %)
No	82 (82 %)

The majority of patients were females (89 %) with a median age of 44 years (range 21–71). There were 67 patients with hormonal contraception with a median time of hormone use of 18 years (range 1–35). In 23 patients hormonal contraceptives were discontinued after presumed diagnosis of FNH. Preoperative liver blood tests were within the normal ranges; only AP and y-GT levels were slightly elevated (AP: 2.5 μkatl/l, reference range 1.1 and y-GT: 1.4 μmmol/l, reference range: 1.2).

The leading indication for liver resection was symptoms with abdominal discomfort, in 46 patients (46 %), whereas FNH were found as an “incidentaloma” in 54 asymptomatic patients (54 %). In most patients, there was more than one indication for surgery, including abdominal discomfort (*n* = 46 patients, 40.7 %), uncertainty of diagnosis/assumption of malignancy in preoperative imaging (*n* = 54 patients, 47.8 %), including cancer history in 18 of the 54 patients (11.5 %) and tumor enlargement/jaundice with a rate of growth of >0.5 cm/year or 2–3 cm in comparison with initial size in 13 patients (11.5 %) (Fig. 1).

The mean tumor enlargement of the 13 patients was 2.4 cm (range 0.5–3.0). In 9 patients jaundice with compression of vascular and biliary structures were observed. Of 18 patients with a history of cancer, patients had been diagnosed with colorectal cancer (*n* = 2; 11.11 %), gynecological cancer (breast cancer: *n* = 4; 22.22 %; ovarian or endometrial cancer: *n* = 5, 27.78 %), urogenital cancer (renal cancer *n* = 2; 11.11 %), pancreatic cancer (*n* = 1; 5.56 %), skin cancer (*n* = 2; 11.11 %) or others (*n* = 2; 11.11 %).

Regarding the pathological characteristics, median tumor diameter was 5.9 cm (range 2–17) with a number of lesions ranging between 1 and 7. In most patients (76 %) tumor distribution was unilobular. Final histology revealed classical FNH in 80 % and non-classical FNH in 20 %. Of these 20 non-classical FNH 11 were classified from local pathology as atypical form, 4 as mixed forms and 5 as teleangietasic form. Histological examination showed a proliferation of the bile duct in 67 specimen (67 %), with central scars being observed in 69 (69 %) patients.

### Diagnostics

Abdominal ultrasound was performed in all patients as first approach, with FNH being correctly diagnosed in 31 % by this imaging modality. There was no trend evident towards a higher sensitivity or specificity of abdominal ultrasound for FNH over the investigated time periods. 24 Patients were diagnosed incorrectly by ultrasound, with a malignant tumor suspected in 10 %, hemangioma in 8 % and adenoma in 6 %. Additional cross-sectional imaging was performed in 99 of these patients, with only one patient being operated following ultrasound for acute appendicitis (*n* = 1) (Table 2).

**Table 2:** Rate of diagnoses and different imaging modalities of our study population during the different time periods

Modality/diagnosis	*N*	Total	1993–1997 (1)	1998–2002 (2)	2003–2007 (3)	2008–2012 (4)
Sonography	100	100 (100 %)	20 (100 %)	31 (100 %)	38 (100 %)	11 (100 %)
Correct		31 (31 %)	8 (40 %)	6 (19.4 %)	14 (36.8 %)	3 (27.3 %)
Uncertain		45 (45 %)	8 (40 %)	18 (58.1 %)	17 (44.7 %)	2 (18.2 %)
Incorrect		24 (24 %)	4 (20 %)	7 (22.6 %)	7 (18.4 %)	6 (54.5 %)
CT	78	78 (100 %)	17 (100 %)	24 (100 %)	31 (100 %)	6 (100 %)
Correct		33 (42.3 %)	9 (52.9 %)	11 (45.8 %)	11 (35.5 %)	2 (33.3 %)
Uncertain		28 (35.9 %)	5 (29.4 %)	6 (25 %)	14 (45.2 %)	3 (50 %)
Incorrect		17 (21.8 %)	3 (17.6 %)	7 (29.2 %)	6 (19.4 %)	1 (16.7 %)
MRI	73	73 (100 %)	12 (100 %)	19 (100 %)	31 (100 %)	11 (100 %)
Correct		43 (58.9 %)	9 (75 %)	11 (57.9 %)	17 (54.8 %)	6 (54.5 %)
Uncertain		22 (30.1 %)	2 (16.7 %)	6 (31.6 %)	11 (35.5 %)	3 (27.3 %)
Incorrect		8 (11 %)	1 (8.3 %)	2 (10.5 %)	3 (9.7 %)	2 (18.2 %)

CT and MRI were performed as first cross-sectional imaging approaches in 76 and 23 patients, respectively, of which 31 patients (41 %) and 12 patients (52 %) were diagnosed correctly with FNH. To increase the degree of diagnostic certainty, a second sectional imaging modality was chosen in 52 patients.

Of the 45 patients with unclear diagnoses in CT as first cross imaging, MRI showed correct diagnoses in 17 patients. Of the 10 patients with unclear diagnoses in MRI as first cross-sectional imaging, none patient received a CT (Fig. 1). In totally, FNH was diagnosed correctly in 17 of 35 (49 %) cases by MRI, in which CT had lead to incorrect or uncertain diagnosis. Both modalities results are significantly different from one another (*p* = 0.006) (Table 3). Interestingly, there was a trend towards a higher rate of correct diagnosis for atypical FNH, when compared to typical FNH in both CT and MRI (Table 4).

**Table 3:** Comparison in finding preoperative correct vs. uncertain/incorrect diagnosis using CT and MRI. Values were illustrated as frequency (%) from total examined patient collective (*n* = 52)

Variables	Total	MRI uncertain/incorrect	MRI correct
CT uncertain/incorrect	35 (100 %) (67.3 %) (67.3 %)	18 (51.4 %) (90 %) (34.6 %)	17 (48.6 %) (53.1 %) (32.7 %)
CT correct	17 (100 %) (32.7 %) (32.7 %)	2 (11.8 %) (10 %) (3.8 %)	15 (88.2 %) (46.9 %) (28.8 %)
	52 (100 %) (100 %) (100 %)	20 (38.5 %) (100 %) (38.5 %)	32 (61.5 %) (100 %) (61.5 %)
*p* value			0.006

**Table 4:** Comparison of CT and MRI for finding the distinct postoperative histopathological FNH forms

Imaging diagnosis	Total	Classical FNH	Non-classical FNH
CT uncertain/incorrect	45 (100 %) (57.7 %) (57.7 %)	40 (88.9 %) (61.5 %) (51.3 %)	5 (11.1 %) (38.5 %) (6.4 %)
CT correct	33 (100 %) (42.3 %) (42.3 %)	25 (75.8 %) (38.5 %) (32.1 %)	8 (24.2 %) (61.5 %) (10.3 %)
Total CT certainty	78 (100 %) (100 %) (100 %)	65 (83.3 %) (100 %) (83.3 %)	13 (16.7 %) (100 %) (16.7 %)
MRI uncertain/incorrect	30 (100 %) (41.1 %) (41.1 %)	26 (86.7 %) (44.8 %) (35.6 %)	4 (13.3 %) (26.7 %) (5.5 %)
MRI correct	43 (100 %) (58.9 %) (58.9 %)	32 (74.4 %) (55.2 %) (43.8 %)	11 (25.6 %) (73.3 %) (15.1 %)
Total MRI certainty	73 (100 %) (100 %) (100 %)	58 (79.5 %) (100 %) (79.5 %)	15 (20.5 %) (100 %) (20.5 %)

Regarding the different time intervals, there were no statistical significant differences in sensitivity or specificity of abdominal CT or MRI (Table 2).

With regard to the median tumor size of our patients (<5.5 vs. >5.5 cm), there were no significant differences observed in preoperative accuracy by CT and MRI over the observation period (CT, *p* = 0.6; MRI, *p* = 0.3). However, regarding the different time periods, patients with FNH >5.5 cm had a trend for a higher preoperative accuracy in the last years (period 1–3 vs. period IV*, p* = 0.07).

^99^Tc hepatobiliary scintigraphy (until 2005) and percutaneous fine-needle biopsy (FNB) were performed as additional diagnostic approaches to cross-sectional imaging in 26 and 21 patients, respectively. Correct diagnosis of FNH was achieved by FNB in 10 of 21 cases, whereas 11 FNB were misdiagnosed as adenoma (*n* = 5), metastasis and/or assumption of hepatocellular carcinoma (*n* = 3) or inconclusive results (*n* = 3).

### Operative and perioperative outcomes

Median duration of the operation was 163 min (range 70–445). The majority of patients (68 %) underwent minor resections, e.g. atypical resection or (bi)segmentectomy, of which 14 patients (22 %) were operated laparoscopically. In 32 patients (32 %) major liver resections (hemihepatectomy or extended hemihepatectomy) were performed. Temporal occlusion of the hepatoduodenal ligament (Pringle maneuver) was performed in 60 patients. Transfusion of RBCC or FFP was indicated intraoperatively in 9 patients (9 %). Median length of stay at ICU was 1.2 days (range 0–6), with a median duration of hospital stay of 16 days (range 1–50). Perioperative morbidity was observed in 19 patients (19 %), perioperative mortality in 0 %. Minor complications (grade I, *n* = 4; grade II, *n* = 7) were observed in 11 patients, major complications in 8 patients (grade IIIa, *n* = 5; grade IIIb, *n* = 2; grade IV, *n* = 1) (Table 5).

**Table 5:** Operative and perioperative details of our study population (*n* = 100 patients)

Variables	*N* (%) or median (range)
Extent of resection	
Minor	68 (68 %)
Laparascopic approach	14 (14 %)
Major	32 (32 %)
Transfusion intraoperative	
Substitution	9 (9 %)
Transfusion erythrocyte concentrations	0.23 (0–6)
Transfusion fresh frozen plasma	0.2 (0–8)
Operating time (min)	163 (70–445)
Hospital stay (days)	16 (1–50)
Intensive care unit (days)	1.2 (0–6)
Complications	
Total complications	19 (19 %)
Complication grade (according to Clavien–Dindo)	
I	4
II	7
IIIa	5
IIIb	2
IVa	1
IVb	0
V	0

The length of ICU hospital stay (>1 day; *p* = 0.02), the type of surgical procedure (open procedure; *p* = 0.027, major resection; *p* = 0.02) and tumor distribution (unilobular; *p* = 0.04) were associated with statistical significant increased rates of complications. Other demographic, clinicopathological, tumor- and procedure-specific factors like age (>44 vs. <44 years, *p* = 0.3), gender (male vs. female, *p* = 0.09), length of intensive care unit stay (<14 vs. >14 days, *p* = 0.07), history of cancer (yes vs. no, *p* = 0.1), transfusion (yes vs. no, *p* = 0.2) and aminopyrine breath test (ABT) (<0.6 vs. >0.6, *p* = 0.6) showed no statistical significance for increased complications.

### Quality of life

Questionnaires evaluating QoL were sent to all patients, of which 57 % (57 patients) of all patients sent questionnaires back for further retrospective analysis. They were completed at a median of 124 months (range 6–229 months) postoperatively.

Most patients reported an overall improvement of their general health after surgery (“very good–excellent”: preoperatively 47.4 % vs. postoperatively 68.1 %; *p* = 0.015). 50 patients (88 %) were satisfied with their decision to have undergone surgery and would make the same decision again. Preoperative symptoms included abdominal pain (*n* = 36, 63 %), nausea/vomitus (*n* = 14, 25 %), fatigue (*n* = 21, 37 %), tenderness (*n* = 29, 51 %), loss of weight (*n* = 13, 23 %) and decreased/loss of appetite (*n* = 13, 23 %). The duration of symptoms was reported to be up to 6 months, 1 and 3 years preoperatively in 65, 17.5 and 17.5 %, respectively (Table 6). Reasons for surgery included impaired lifestyle (*n* = 7, 12 %), fear of complications without therapy (*n* = 11, 19 %), concerns for underlying malignancy (*n* = 47, 82 %), doctor’s recommendation (*n* = 26, 46 %) or general worries (*n* = 13, 23 %).

**Table 6:** Preoperative symptoms and duration of symptoms before liver surgery of our study collective (*n* = 57 patients)

Variables	*N* (%)
Nausea/vomiting	
None	43 (75.4)
Mild/moderate	12 (21.1)
Severe	1 (1.8)
Extreme	1 (1.8)
Loss of weight	
None	44 (77.2)
Mild/moderate	7 (12.3)
Severe	2 (3.5)
Extreme	4 (7)
Tenderness	
None	28 (49.1)
Mild/moderate	20 (35.1)
Severe	5 (8.8)
Extreme	4 (7)
Fatigue	
None	36 (63.2)
Mild/moderate	9 (15.8)
Severe	8 (14)
Extreme	4 (7)
Decreased appetite	
None	44 (77.2)
Mild/moderate	7 (12.3)
Severe	3 (5.3)
Extreme	3 (5.3)
Duration of symptoms	
<3 months	26 (45.6)
3–6 months	11 (19.3)
6–9 months	4 (7)
9–12 months	6 (10.5)
1–3 years	3 (5.3)
>3 years	7 (12.3)

Regarding the general QoL, 49.6 % of patients reported a “much better” or “somewhat better” overall QoL after surgery, with improvements in both physical and mental health. In particular, the proportion of patients reporting “a little’’ or ‘‘a lot’’ of limitations with moderate activity decreased from 44.1 % preoperatively to 29.4 % postoperatively (*p* < 0.001). Another domain was the mental health status of patients, which also improved after surgery. Postoperatively, a larger proportion of patients reported to feel calm and peaceful ‘‘at all times’’ or ‘‘most times’’ (preoperatively 42.6 % vs. postoperatively 69.1 %; *p* = 0.005). Patients also reported improvements in energy levels, with more patients reporting lots of energy ‘‘at all times’’ or ‘‘most times’’ (preoperatively 45.6 % vs. postoperatively 66.2 %; *p* < 0.0016). Fewer patients noted depressed moods postoperatively (preoperatively 12.7 % vs. postoperatively 5.9 %; *p* = 0.04). Fewer patients reported social impairment postoperatively (physical health or emotional problems interfere with social life ‘‘all the time’’ or ‘‘often’’: preoperatively 21.9 % vs. postoperatively 7.3 %; *p* < 0.001).

Of 36 patients, whoreported pain preoperatively, 8 patients (14 %) suffered from mild pain, whereas 28 patients (49 %) from moderate to extreme pain (Table 7). Mean preoperative pain score level did not change 1 month postoperatively (1.33 ± 0.17, *p* = 0.6). Mean pain levels decreased significantly over time from 1.49 (±0.18) preoperatively to 0.82 (±0.17) and 0.35 (±0.09) at 6 months and 1 year postoperatively (*p* = 0.003 and *p* < 0.001). Pain scores decreased over time with no significant differences noted between laparascopic and open approaches. However, patients undergoing laparoscopic liver resection reported 2.3-fold more frequently about an improvement of their life quality postoperatively, when compared to patients undergoing open operation (OR 5.8; 95 % CI 1.1–31.1; *p* = 0.03).

**Table 7:** Comparison of preoperative and postoperative pain level of our study population

Pain level	Preoperative	First month postoperative	6 months postoperative	1 year postoperative	*p* value
Pain level (%)					
0 (none)	21 (36.8 %)	23 (40.4 %)	25 (43.9 %)	43 (75.4 %)	
1 (mild)	8 (14 %)	9 (15.8 %)	21 (36.8 %)	9 (15.8 %)	
2 (moderate)	12 (21.1 %)	12 (21.1 %)	7 (12.3 %)	4 (7 %)	
3 (severe)	11 (19.3 %)	9 (15.8 %)	4 (7 %)	1 (1.8 %)	
4 (extreme)	5 (8.8 %)	4 (7.0 %)	0 (0 %)	0 (0 %)	
Mean ± standard deviation	1.49 ± 0.18	1.33 ± 0.17	0.82 ± 0.12	0.35 ± 0.09	<0.001

Analyzing the QoL of all patients undergoing liver resection for FNH revealed a benefit especially for patients with preoperative symptoms. Patients with “moderate-to-extreme” symptoms were more likely to report an improvement in general QoL postoperatively, when compared to patients with no or only mild symptoms preoperatively. In particular, patients who reported on ‘‘moderate-to-extreme’’ pain preoperatively were more likely to report an improvement in QoL postoperatively than patients who initially reported no or mild pain (OR, 3.6; 95 % CI 1.1–11.4; *p* = 0.02). Furthermore, the preoperative presence of ‘‘moderate to extreme’’ tenderness (OR, 3.5; 95 % CI 1.1–10.5; *p* = 0.03), ‘‘moderate-to-extreme’’ decreased appetite (OR, 6.3; 95 % CI 1.2–31.5; *p* = 0.02), ‘‘moderate-to-extreme’’ fatigue (OR, 3.5; 95 % CI 1.0–11.8; *p* = 0.04) such as symptoms >9 months (OR, 4.7; 95 % 1.3–16.4; *p* = 0.01) were all strongly associated with improved QOL postoperatively (Table 8).

**Table 8:** Analysis of factors associated with improved overall quality of life after hepatic surgery of FNH

Variables	Odds ratio (95 % CI)	*p* value
Abdominal pain		
Moderate to extreme	3.6 (1.1–11.4)	0.024*
Tenderness		
Moderate to extreme	3.5 (1.1–10.5)	0.03*
Nausea		
Moderate to extreme	2.5 (0.1–0.7)	0.1
Decreased appetite		
Moderate to extreme	6.3 (1.2–31.5)	0.025*
Fatigue		
Moderate to extreme	3.5 (1.0–11.8)	0.04*
Type of surgery		
Laparascopic approach	5.8 (1.1–31.1)	0.03*
Minor resection	0.9 (0.2–3.2)	0.9
Complications		
No complications	2.6 (0.5–14.4)	0.2
Age <40 years	1.4 (0.4–4.4)	0.5
Female gender	0.5 (0.1–3.1)	0.45
Tumor size >50 mm	1.8 (0.6–5.3)	0.2
BMI <25 (kg/m^2^)	1.7 (0.6–5.1)	0.29
Hospital stay <16 days	1.1 (0.3–3.2)	0.9
Unilobular tumor distribution	2.4 (0.5–10.3)	0.2
Classical FNH	0.4 (0.1–1.4)	0.12
Symptoms >9 months	4.7 (1.3–16.4)	0.01*
Uncertainty of malignancy	3.5 (0.6–18.3)	0.1
Fear of complications from liver disease	1.8 (0.5–7.0)	0.3

*BMI* body mass index, *FNH* focal nodular hyperplasia

* Statistically significant

Other demographic, clinicopathological, tumor- and procedure-specific factors, such as tumor size, extent of resection, operative approach, complications in the perioperative period, uncertainty of malignancy or fear of complications from liver disease had no impact on QOL after recovery.

## Discussion

This study represents one of the largest retrospective analyses, examining patients undergoing liver resection for FNH to date. Despite remarkable advantages in diagnostic modalities within the last years, there remains a considerable proportion of misleading preoperative diagnosis, especially in patients with atypical forms of FNH and history of cancer. Reasons for surgery included abdominal discomfort, uncertainty of diagnosis/malignancy and tumor enlargement/jaundice. Our results clearly indicate that liver resection can be performed safely with major complications evident in only 8 % and no mortality. Surgery for FNH is associated with a high patient satisfaction and in our study improved QoL, especially in symptomatic patients.

There is an emerging interest in managing incidental findings in the liver and pancreas [3–5, 7–9, 37]. Among cross-sectional imaging, MRI is considered the most reliable imaging method for classifying incidental liver lesions, especially after recent improvements, e.g. the introduction of the new T1-positive liver specific contrast agent, gadoxetic acid (Gd-EOB-DTPA, Primovist or Evosit, Bayer Schering Pharma, Germany) [13–18]. However, even after combining different non-invasive imaging modalities, e.g. CT and MRI, and invasive percutaneous fine-needle biopsy, diagnostic uncertainty remains in up to 40 %. In accordance with the current literature, we noted incorrect/uncertain findings on CT and MRI in 58 and 41 %, respectively, with 40 % remaining incorrect/uncertain after combined both diagnostic approaches [10, 11, 20]. When the CT was incorrect/uncertain, running an additional MRI did not help establish a more correct preoperative diagnosis in our study. There were no statistical significant improvements with regard to the percentage of correct diagnosis over the investigated time periods. Our data show a non significant decrease in surgical procedures between 1992 and 2012 in patients with histologically proven diagnosis of FNH. The apparent decrease of carried out surgical procedures does not necessarily represent a decrease in incidence of FNH, but only a decrease of patients who underwent surgical procedures for FNH. The reason for the decrease of surgical interventions in FNH patients lies, in our opinion, in the more accurate and reliable diagnostic modalities. Of note, percentage of incorrect/uncertain diagnosis by CT and MRI is likely invalid high, as the patients’ collective does not represent all patients with FNH, but only patients undergoing surgery.

Percutaneous fine-needle biopsy represents another diagnostic possibility in the workflow for uncertain liver lesions. However, its significance is discussed controversially, as it is associated with an increased bleeding risk in hypervascularized lesions and a risk of peritoneal seeding in case of malignancy [2–7, 19–21]. Further, tumor biopsy is associated with a low diagnostic sensitivity, with only 30–45 % of all biopsy being consistent with the histology of surgical preparations [2, 3, 21]. This could be confirmed in our patients’ cohort, where only in 10 of 21 fine-needle biopsies (47 %) were confirmed by histology postoperatively. Apart from operative management strategies of symptomatic FNH, percutaneous radiological modalities have to be considered and include arterial embolization and radiofrequency ablation (RFA), which have been published in a few case series [10, 38–40]. However, up to date no randomized controlled trials exist comparing the outcome of surgical resection with percutaneous techniques. Of interest, the major limitation of these interventional techniques is the lack of post-procedural histology. Therefore, these techniques should only be applied if a definitive diagnosis of FNH could be secured by preoperative imaging and biopsy-derived diagnosis [10].

Most incidentalomas are likely benign without or little clinical significance. Committees have recently been formed to manage incidental findings on CT and consensus guidelines try to classify patients groups of high risk with hepatic and pancreatic lesions [8, 9, 28, 37]. Factors like age (>40 years), history of malignancy, hepatic malignant risk factors as well as hepatic dysfunction with or without symptoms help to define patients of considerably high risk for liver malignancies [8, 9, 37]. In accordance, surgery was performed in our cohort because of uncertainty of diagnosis/malignancy in 41 patients, cancer history in 18 patients and tumor enlargement/jaundice with a rate of growth of >0.5 cm/year or 2–3 cm in comparison with initial size in 13 patients.

Certainly, special guidelines for benign liver lesions are needed to balance cost-intensive long-term follow ups by MRI against the risk of unnecessary operations associated with perioperative complications. We need to take in account not only improvements in radiological findings; due to ongoing improvements in liver surgery with decreased perioperative complications even major hepatic resections can be safely performed in experienced hepatobiliary centers [19, 22, 29, 30, 41–43]. Based on our results with low perioperative major complications (8 %) and no mortality, this might influence the process making a decision in these patients. In contrast to asymptomatic patients diagnosed with incidentalomas of the liver, 46 patients underwent liver surgery because of abdominal discomfort with nonspecific symptoms (nausea, fatigue, decreased appetite, etc.) or pain. Although the evaluated symptoms represent typical complaints of patients with benign hepatic tumors, they are admittedly not specific for FNH. However, the symptoms listed in our questionnaire were chosen in support of similar studies evaluating QoL in patients with surgery for benign hepatic tumors and therefore seem suited to evaluate QoL postoperatively [30, 31]. Abdominal pain as indication for surgery can be challenging, as it is difficult to provide assurances that symptoms will improve after surgery [22, 30]. Therefore, liver resection should be considered only after exclusion of other causes for abdominal symptoms and if a relief of symptoms is expected after surgery. Only a few reports on QoL improvements after liver resection of benign liver tumors exist, with no sufficient data for FNH available [22, 30, 31, 41, 43].

Beside a decreased complication rate after liver surgery, long-term outcome represents an important factor for evaluating patients for surgery. Our study provides first evidence that QoL might improve in patients with symptomatic FNH after liver resection.

The mean patient self-reported pain levels had significantly decreased over time. In addition to pain and other preoperative symptoms, several further QOL domains including physical health, mental/social/emotional health such as general health were evaluated. We noted improvements in a wide range of QOL domains with 20 % of our patients reporting a significant improvement of general health after liver resection. Furthermore, significant improvements were noted not only in physical activities, but also in social and mental health. These improvements resulted in greater work productivity, increased energy level and better social functioning. These findings together with the fact that 88 % of patients were generally satisfied and would undergo liver surgery again seem to prove a positive effect of liver surgery on daily life of the patients.

Regarding the different types of surgical approaches, e.g. laparascopic vs. open surgery, there were no statistically significant differences with regard to reported pain levels over time. This is in contrast to previous studies by Kneurtz et al., who noted markedly better pain scores after laparascopic surgery 6 and 12 months after liver resection [31]. This controversy might be due to the fact that only patients scheduled for minor resections were evaluated for laparoscopic approaches in our cohort. In accordance with similar studies, patients undergoing laparoscopic liver resection reported a postoperative improvement of QoL of 2.3-fold compared to patients undergoing open surgery [31]. Even though an interpretation of the QoL data is limited by its retrospective character, these results give us a strong impression of patient-related outcome after resection of benign liver tumors and illustrate the benefits of surgery from patient’s point of view.

The current study has several limitations that should be considered. First, data were collected and analyzed retrospectively with a considerable time period between surgical procedure and patients’ interview with a response rate of approximately 60 %. In this context, the collection of data may be influenced by recall bias. For example, a possible concern might be that patients with better operative outcomes (e.g.: reduction of postoperative pain score, no presence of malignant tumor in final postoperative histology, no operative complications) answered the QoL survey more often than patients with inferior postoperative outcome. This bias could lead to an overestimation of the postoperative improvement of QoL in our study. In our study, we found an overall complication rate of 19 % concerning all conducted surgeries in benign hepatic tumors. The patients who answered the QoL questionnaire had an overall complication rate of 16 %, comparably to the overall complication rate. Therefore, if one may consider the complication rate as parameter for negative outcome or negative association of the surgical intervention by the patient, a similar complication rate might contradict the suggested recall bias. Another limitation might be that patients with the pre-existing diagnosis of a malignant tumor might feel more worried by the presence of a malignant tumor in the liver and postoperative benign diagnosis may have brought these patients more relief compared to patients without a cancer history. Patients with a subjective uncertainty of malignancy had a non significant increase in QoL after hepatic surgery for FNH. However, all other patients with preoperative proven diagnosis of a benign tumor nature also showed an insignificant increase of QoL. Therefore, we suggest that the histologically proven certainty of benign tumor nature may play an important role in QoL for patients, who are worried about the potential malignancy of the tumor; however, patients without worries about tumor malignancy also profited from the operation in our study.

## Conclusions

This study demonstrates that liver resection for benign liver tumors can be performed safely in specialized hepatobiliary centers. Despite improvements in diagnostic modalities, there remain liver lesions that cannot be specified reliably by imaging. In case of uncertain diagnosis, especially in patients with a history of malignancy or suspected hepatocellular adenoma, marked tumor enlargement and/or jaundice, surgical resection should be considered. In case of symptomatic liver lesions, surgical resection should only be indicated in patients with tumor-specific symptoms. Regarding our results, surgery for FNH is associated with marked improvements in patient-reported pain scores as well as other QoL domains. Patients with significant preoperative symptoms show the most benefit from surgical intervention.

## Additional files

10.1186/s40001-015-0181-x Quality of life survey in German
10.1186/s40001-015-0181-x Quality of life survey in English

## Abbreviations

FNH: focal nodular hyperplasia
QoL: quality of life
CT: computed tomography
TSTC: too small to characterize
HCC: hepatocellular carcinoma
MRI: magnetic resonance imaging
ERCP: endoscopic retrograde cholangio-pancreatography
ALAT: alanine aminotransferase
ASAT: aspartat aminotransferase
y-GT: y-glutamyltransferase
AP: alkaline phosphatase
RBCC: red blood cell concentrate
FFP: fresh frozen plasma
ICU: intensive care unit
CI: confidence interval
OR: odds ratios
SD: standard deviations
FNB: percutaneous fine-needle biopsy
ABT: aminopyrine breath test
cm: centimeter
g: gram
