# Supplementary material for: The value of liver resection for focal nodular hyperplasia: resection yes or no?
Source: Eur J Med Res. 2015 Oct 22;20:86. doi: 10.1186/s40001-015-0181-x (PMC4619214; doi:10.1186/s40001-015-0181-x)
Supplement: Supplementary file 1 — 10.1186/s40001-015-0181-x Quality of life survey in German [file 40001_2015_181_MOESM1_ESM.doc]

**In this survey we like to collect some information concerning your liver operation at the University Hospital of Leipzig. Your information helps us to evaluate your health and the pain development pre- and postoperative.**

**Please answer the following questions on your own by choosing the best applicable answer and confirm the accuracy of your information with your signature.**

**1. General questions:**

Name: _______________ Surname: _______________ Date of Birth:___.___._____

Signature:_______________________

1. Did you take one of the following medications before you got ill? If yes, please specify how often, how long and in which dose you took the medication.
   - ASS number per day: _____ duration: ______ dose:_______
   - Paracetamol number per day: _____ duration: ______ dose:_______
   - Steroids number per day: _____ duration: ______ dose:_______
   - Antidepressants number per day: _____ duration: ______ dose:_______

If your sex is female:

- - Birth Control Pill: name:_______________ , consumed since:_________ until:______

1. Which describes the correct period you suffered of any symptoms before you decided to go to the University Hospital of Leipzig for surgery?
   - < 3 months
   - 3-6 months
   - 6-9 months
   - 9-12 months
   - 1-3 years
   - > 3 years
2. Why did you choose to have the operation? Please select the correct field or complete the field “other”.

- I was affected by the disease in everyday life
- I was afraid of complications caused by my liver disease
- I was afraid the tumor might be malignant
- My doctor recommended the operation
- I had generall worries
- Other: ___________________________________________________________________

**The second part evaluates your health preoperatively**

|  | **Not applicable** | **Somewhat applicable** | **Quite applicable** | **Highly applicable** |
| --- | --- | --- | --- | --- |
| **1. Did you have any trouble with feeding?** |  |  |  |  |
| **2. Did you experience a feeling of satiety too quickly after starting to eat?** |  |  |  |  |
| **3. Did you feel less appetite than before you got ill?** |  |  |  |  |
| **4. Did you lose a lot of weight during the last 3 months before the operation?** |  |  |  |  |
| **5. Did you suffer from nausea or vomiting?** |  |  |  |  |
| **6. Did you suffer from pain in the stomach area?** |  |  |  |  |
| **7. Did you feel impaired in everyday life because of the pain?** |  |  |  |  |
| **8. Did you develop a yellow tone of skin or eyes (jaundice)?** |  |  |  |  |
| **9. Did you suffer from a feeling of pressure in your stomach?** |  |  |  |  |
| **10. Did you suffer from pain in your back?** |  |  |  |  |

| **11. Pain intensity** | **No pain** | **weak pain** | **Medium pain** | **Strong pain** | **Very strong pain** |
| --- | --- | --- | --- | --- | --- |
| preoperative |  |  |  |  |  |

12. Please indicate your current pain level compared to your **intensity of pain before the operation**, with 0 = **No pain**, 10= unbearable pain.

□ 0 □ 1 □ 2 □ 3 □ 4 □ 5 □ 6 □ 7 □ 8 □ 9 □ 10 **No pain** 🡪 unbearable pain

|  | **Not applicable** | **Somewhat applicable** | **Quite applicable** | **Highly applicable** |
| --- | --- | --- | --- | --- |
| **13. Did you feel a lack of energy?** |  |  |  |  |
| **14. Did you feel restricted in everyday life?** |  |  |  |  |
| **15. Did you have problems in social life activities such as hobbies?** |  |  |  |  |
| **16. Did you have to take a rest more often than before?** |  |  |  |  |
| **17. Did you feel tired more often than before?** |  |  |  |  |
| **18. Did you have problems meeting with friends as usual?** |  |  |  |  |
| **19. Did you have problems in telling your friends or family members about your disease?** |  |  |  |  |
| **20. Did you feel more stressed out than before your disease?** |  |  |  |  |
| **21. Did you worry about your future health?** |  |  |  |  |
| **22. Did you worry about your family in the future because of your disease?** |  |  |  |  |
| **23. Did you have financial worries because of your physical condition or the medical treatment?** |  |  |  |  |
| **24. Did you have problems concentrating e.g. during watching television or reading the newspapers?** |  |  |  |  |
| **25. Did you feel more tense than before the disease?** |  |  |  |  |
| **26. Did you feel more depressed than before the disease?** |  |  |  |  |
| **27. Did you feel happy despite the disease?** |  |  |  |  |

In the following some activities of daily living are listed. Did you had any problems while performing these activities because of your health conditions preoperatively? If yes, in which intensity?

|  | **Not applicable** | **Somewhat applicable** | **Quite applicable** | **Highly applicable** |
| --- | --- | --- | --- | --- |
| **28. Performing exhausting activities: e.g. running, lifting heavy weights, practicing exhausting sports** |  |  |  |  |
| **29. Performing moderate activities: e.g. shifting a table, vacuum-cleaning, playing golf** |  |  |  |  |
| **30. Carrying shopping bags** |  |  |  |  |
| **31. Climbing stairs: more than one landing** |  |  |  |  |
| **32. Climbing stairs: one landing** |  |  |  |  |
| **33. Bowing, kneeling down, bending down** |  |  |  |  |
| **34. Walking more than one kilometre** |  |  |  |  |
| **35. Walking more than 500 metres** |  |  |  |  |
| **36. Walk less than 500 metres** |  |  |  |  |
| **37. Bathing or getting dressed** |  |  |  |  |

38. How would you assess your health condition before the operation? Please use school grades!

□ 1 □ 2 □ 3 □ 4 □ 5 □ 6

Very good 🡪 very bad

39. How would you assess your quality of life before the operation? Please use school grades!

□ 1 □ 2 □ 3 □ 4 □ 5 □ 6

Very good 🡪 very bad

**The third part considers your health conditions postoperative.**

|  | **Not applicable** | **Somewhat applicable** | **Quite applicable** | **Highly applicable** |
| --- | --- | --- | --- | --- |
| **1. Did you have any trouble with feeding?** |  |  |  |  |
| **2. Did you experience a feeling of satiety too quickly after starting to eat?** |  |  |  |  |
| **3. Did you feel less appetite than before you got ill?** |  |  |  |  |
| **4. Did you lose a lot of weight during the last 3 months before the operation?** |  |  |  |  |
| **5. Did you suffer from nausea or vomiting?** |  |  |  |  |
| **6. Did you suffer from pain in the stomach area?** |  |  |  |  |
| **7. Did you feel impaired in everyday life because of the pain?** |  |  |  |  |
| **8. Did you develop a yellow tone of skin or eyes (jaundice)?** |  |  |  |  |
| **9. Did you suffer from a feeling of pressure in your stomach?** |  |  |  |  |
| **10. Did you suffer from pain in your back?** |  |  |  |  |

11. Please indicate the level of **intensity of pain postoperative**, where 0 = **No pain**, 10 = **unbearable pain**.

□ 0 □ 1 □ 2 □ 3 □ 4 □ 5 □ 6 □ 7 □ 8 □ 9 □ 10 **No pain** 🡪 **unbearable pain**

|  | **Not applicable** | **Somewhat applicable** | **Quite applicable** | **Highly applicable** |
| --- | --- | --- | --- | --- |
| **12. Did you feel a lack of energy?** |  |  |  |  |
| **13. Did you feel restricted in everyday life?** |  |  |  |  |
| **14. Did you have problems in social life activities such as hobbies?** |  |  |  |  |
| **15. Did you have to take a rest more often than before?** |  |  |  |  |
| **16. Did you feel tired more often than before?** |  |  |  |  |
| **17. Did you have problems meeting with friends as usual?** |  |  |  |  |
| **18. Did you have problems in telling your friends or family members about your disease?** |  |  |  |  |
| **19. Did you feel more stressed out than before your disease?** |  |  |  |  |
| **20. Did you worry about your future health?** |  |  |  |  |
| **21. Did you worry about your family in the future because of your disease?** |  |  |  |  |
| **22. Did you have financial worries because of your physical condition or the medical treatment?** |  |  |  |  |
| **23. Did you have problems concentrating e.g. during watching television or reading the newspapers?** |  |  |  |  |
| **24. Did you feel more tense than before the disease?** |  |  |  |  |
| **25. Did you feel more depressed than before the disease?** |  |  |  |  |
| **26. Did you feel happy despite the disease?** |  |  |  |  |

In the following some activities of daily living are listed. Did you had any problems while performing these activities because of your health conditions postoperatively? If yes, in which intensity?

|  | **Not applicable** | **Somewhat applicable** | **Quite applicable** | **Highly applicable** |
| --- | --- | --- | --- | --- |
| **27. Performing exhausting activities: e.g. running, lifting heavy weights, practicing exhausting sports** |  |  |  |  |
| **28. Performing moderate activities: e.g. shifting a table, vacuum-cleaning, playing golf** |  |  |  |  |
| **29. Carrying shopping bags** |  |  |  |  |
| **30. Climbing stairs: more than one landing** |  |  |  |  |
| **31. Climbing stairs: one landing** |  |  |  |  |
| **32. Bowing, kneeling down, bending down** |  |  |  |  |
| **33. Walking more than one kilometre** |  |  |  |  |
| **34. Walking more than 500 metres** |  |  |  |  |
| **35. Walk less than 500 metres** |  |  |  |  |
| **36. Bathing or getting dressed** |  |  |  |  |

37. How would you assess your health condition after the operation? Please use school grades!

□ 1 □ 2 □ 3 □ 4 □ 5 □ 6

Very good 🡪 very bad

38. How would you assess your quality of life after the operation? Please use school grades!

□ 1 □ 2 □ 3 □ 4 □ 5 □ 6

Very good 🡪 very bad

39. We would like to know your satisfaction with the operation. Please rate by school grades again!

□ 1 □ 2 □ 3 □ 4 □ 5 □ 6

Very good 🡪 very bad

40. Please specify the development of pain in the different post operative periods as listed below:

| Pain intensity | **No pain** | **Weak pain** | **Moderate pain** | **Strong pain** | **Very strong pain** |
| --- | --- | --- | --- | --- | --- |
| **Directly postoperative** |  |  |  |  |  |
| **1 month postoperative** |  |  |  |  |  |
| **6 months postoperative** |  |  |  |  |  |
| **1 year postoperative** |  |  |  |  |  |

**Space for your remarks**

**The fourth part considers your current health condition.**

1. Please choose a number describing your current **intensity of pain**, where 0= **No pain**, 10= unbearable pain.

□ 0 □ 1 □ 2 □ 3 □ 4 □ 5 □ 6 □ 7 □ 8 □ 9 □ 10 **No pain** 🡪 unbearable pain

2. How would you assess your current health condition? Please use school grades!

□ 1 □ 2 □ 3 □ 4 □ 5 □ 6

Very good 🡪 very bad

3. How would you assess your current quality of life? Please use school grades!

□ 1 □ 2 □ 3 □ 4 □ 5 □ 6

Very good 🡪 very bad.

4. Last but not least we would like to know to what extent each of the following statements applies to you. Please tick the relevant answer!

|  | **Completely agree** | **Generally agree** | **Not applicable to a large extent** | **Not correct at all** |
| --- | --- | --- | --- | --- |
| **I get sick faster than others.** |  |  |  |  |
| **I'm as healthy as any other person in my environment.** |  |  |  |  |
| **I expect my health condition will deteriorate.** |  |  |  |  |
| **I am in the best of health.** |  |  |  |  |
