# Supplementary material for: The value of liver resection for focal nodular hyperplasia: resection yes or no?
Source: Eur J Med Res. 2015 Oct 22;20:86. doi: 10.1186/s40001-015-0181-x (PMC4619214; doi:10.1186/s40001-015-0181-x)
Supplement: Supplementary file 2 — 10.1186/s40001-015-0181-x Quality of life survey in English [file 40001_2015_181_MOESM2_ESM.doc]

**In diesem Fragebogen geht es um die Erfassung einiger Daten vor und nach Ihrer Leberoperation wegen gutartiger Lebertumore an der Uniklinik Leipzig. Ihre Angaben ermöglichen es uns, Ihren Gesundheitszustand und Ihre Schmerzerfahrung vor und nach der Operation zu beurteilen.**

**Bitte beantworten Sie die Fragen selbstständig, indem Sie die auf Sie am besten zutreffende Antwort ankreuzen und bestätigen die Richtigkeit ihrer Angaben mit Ihrer Unterschrift.**

**1. Allgemeiner Teil**

Name: _______________ Vorname: _______________ Geburtsdatum:___.___._____

Unterschrift:_______________________

1. **Nahmen Sie vor Ihrer Lebererkrankung eines der folgenden Medikamente?** Falls ja, geben Sie bitte an, wie oft, wie lange und in welcher Dosierung es eingenommen wurde.
   - **ASS** Anzahl: Dauer: Dosierung:
   - **Paracetamol** Anzahl: Dauer: Dosierung:
   - **Steroide** Anzahl: Dauer: Dosierung:
   - **Antidepressiva** Anzahl: Dauer: Dosierung:

Bei Frauen:

- **Antibabypille** Name:_______________ , Einnahme seit:_________ bis______

1. **Wie lange vor Ihrer Operation an der Uniklinik Leipzig hatten sie schon Beschwerden? Bitte kreuzen Sie das zutreffende Feld an!**
   - < 3 Monate
   - 3-6 Monate
   - 6-9 Monate
   - 9-12 Monate
   - 1-3 Jahre
   - > 3 Jahre
2. **Warum haben Sie sich für die OP entschieden? Kreuzen Sie bitte das entsprechende Feld an oder ergänzen Sie unter „Sonstiges“.**

- Ich fühlte mich durch die Krankheit im Alltag beeinträchtigt
- Ich hatte Angst vor Komplikationen aufgrund der Lebererkrankung
- Ich war mir nicht sicher, ob der Tumor nicht doch bösartig ist
- Mein Arzt hat mir zur OP geraten
- Ich hatte generell Angst und wollte deshalb eine OP
- Sonstiges: _____________________________________________________________________

**Im zweiten Teil des Fragebogens geht es um Ihren Zustand vor der Operation.**

|  | **Gar nicht zutreffend** | **Etwas zutreffend** | **Ziemlich zutreffend** | **Sehr stark zutreffend** |
| --- | --- | --- | --- | --- |
| **1. Hatten Sie Probleme beim Essen?** |  |  |  |  |
| **2. Hatten Sie schnell ein Völlegefühl beim Essen?** |  |  |  |  |
| **3. Hatten Sie weniger Appetit als vor der Erkrankung?** |  |  |  |  |
| **4. Hatten Sie in den letzten 3 Monaten vor ihrer OP stark an Gewicht verloren?** |  |  |  |  |
| **5. Litten Sie unter Übelkeit/Erbrechen?** |  |  |  |  |
| **6. Hatten Sie Schmerzen im Bauch?** |  |  |  |  |
| **7. Fühlten Sie sich durch ihre Schmerzen im Alltag beeinträchtigt?** |  |  |  |  |
| **8. Hatten Sie gelblich verfärbte Haut oder Augen?** |  |  |  |  |
| **9. Hatten Sie ein Druckgefühl im Bauch?** |  |  |  |  |
| **10. Hatten Sie Rückenschmerzen?** |  |  |  |  |

| 11. Schmerzstärke | **Kein Schmerz** | **Schwacher Schmerz** | **Mittlerer Schmerz** | **Starker Schmerz** | **Sehr starker Schmerz** |
| --- | --- | --- | --- | --- | --- |
| Vor der Operation |  |  |  |  |  |

12. Bitte kreuzen Sie in dieser Skala den Punkt an, der Ihrer **Schmerzintensität vor der OP** entsprach, wobei 0= kein Schmerz, 10= unerträglicher Schmerz bedeutet.

□ 0 □ 1 □ 2 □ 3 □ 4 □ 5 □ 6 □ 7 □ 8 □ 9 □ 10 keine Schmerzen 🡪 unerträglich

|  | **Gar nicht zutreffend** | **Etwas zutreffend** | **Ziemlich zutreffend** | **Sehr stark zutreffend** |
| --- | --- | --- | --- | --- |
| **13. Hatten Sie weniger Energie als üblich?** |  |  |  |  |
| **14. Waren Sie bei Ihrer Arbeit oder anderen tagtäglichen Beschäftigungen eingeschränkt?** |  |  |  |  |
| **15. Waren Sie bei Ihren Hobbys oder anderen Freizeitaktivitäten eingeschränkt?** |  |  |  |  |
| **16. Mussten Sie sich häufiger als vor der Erkrankung ausruhen?** |  |  |  |  |
| **17. Waren Sie häufiger müde als vor der Erkrankung?** |  |  |  |  |
| **18. Hatten Sie Probleme dabei, sich wie üblich mit Freunden oder der Familie zu treffen?** |  |  |  |  |
| **19. Hatten Sie Probleme damit, mit Freunden oder Ihrer Familie über Ihre krankheitsbedingten Gefühle zu sprechen?** |  |  |  |  |
| **20. Fühlten Sie sich gestresster als vor der Erkrankung?** |  |  |  |  |
| **21. Machten Sie sich Gedanken über Ihre Gesundheit in der Zukunft?** |  |  |  |  |
| **22. Machten Sie sich Sorgen über Ihre Familie in der Zukunft aufgrund Ihrer Erkrankung?** |  |  |  |  |
| **23. Hat Ihr körperlicher Zustand oder Ihre medizinische Behandlung Sie in finanzielle Schwierigkeiten gebracht?** |  |  |  |  |
| **24. Hatten Sie Schwierigkeiten, sich auf etwas zu konzentrieren, z.B. auf die Zeitung oder den Fernseher?** |  |  |  |  |
| **25. Fühlten Sie sich angespannter als vor der Erkrankung?** |  |  |  |  |
| **26. Fühlten Sie sich niedergeschlagener als vor der Erkrankung?** |  |  |  |  |
| **27. Waren Sie trotz ihrer Erkrankung glücklich?** |  |  |  |  |

Im Folgenden sind Tätigkeiten beschrieben, die Sie eventuell an einem normalen Tag ausüben. Waren sie durch Ihren Gesundheitszustand **vor der OP** bei diesen Tätigkeiten eingeschränkt? Wenn ja, wie stark?

|  | **Gar nicht einge- schränkt** | **Etwas einge- schränkt** | **Ziemlich einge- schränkt** | **Sehr einge- schränkt** |
| --- | --- | --- | --- | --- |
| **28. Anstrengende Tätigkeiten, z.B. Schnell laufen, schwere Gegenstände heben, anstrengenden Sport treiben** |  |  |  |  |
| **29. Mittelschwere Tätigkeiten z.B. einen Tisch verschieben, staubsaugen, Golf spielen** |  |  |  |  |
| **30. Einkaufstaschen tragen oder heben** |  |  |  |  |
| **31. Mehrere Treppenabsätze steigen** |  |  |  |  |
| **32. Einen Treppenabsatz steigen** |  |  |  |  |
| **33. Sich beugen, knien, bücken** |  |  |  |  |
| **34. Mehr als 1 Kilometer zu Fuß gehen** |  |  |  |  |
| **35. Über 500m zu Fuß gehen** |  |  |  |  |
| **36. Weniger als 500m zu Fuß gehen** |  |  |  |  |
| **37. Sich baden oder anziehen** |  |  |  |  |

38. Wie würden Sie Ihren Gesundheitszustand **vor** der OP einschätzen, wenn Sie Schulnoten vergeben?

□ 1 □ 2 □ 3 □ 4 □ 5 □ 6

ausgezeichnet 🡪 Sehr schlecht

39. Wie würden Sie insgesamt Ihre Lebensqualität **vor** der OP einschätzen,wenn Sie Schulnoten vergeben?

□ 1 □ 2 □ 3 □ 4 □ 5 □ 6

ausgezeichnet 🡪 Sehr schlecht

**Im dritten Teil des Fragebogens geht es um Ihren Zustand nach der Operation.**

|  | **Gar nicht zutreffend** | **Etwas zutreffend** | **Ziemlich zutreffend** | **Sehr stark zutreffend** |
| --- | --- | --- | --- | --- |
| **1. Hatten Sie Probleme beim Essen?** |  |  |  |  |
| **2. Hatten Sie schnell ein Völlegefühl beim Essen?** |  |  |  |  |
| **3. Hatten Sie weniger Appetit als vor der OP?** |  |  |  |  |
| **4. Hatten Sie in den letzten 3 Monaten nach ihrer OP stark an Gewicht verloren?** |  |  |  |  |
| **5. Litten Sie unter Übelkeit/Erbrechen?** |  |  |  |  |
| **6. Hatten Sie Schmerzen im Bauch?** |  |  |  |  |
| **7. Fühlten Sie sich durch ihre Schmerzen im Alltag beeinträchtigt?** |  |  |  |  |
| **8. Hatten Sie gelblich verfärbte Haut oder Augen?** |  |  |  |  |
| **9. Hatten Sie ein Druckgefühl im Bauch?** |  |  |  |  |
| **10. Hatten Sie Rückenschmerzen?** |  |  |  |  |

11. Kreuzen Sie bitte erneut die Ihnen entsprechende Schmerzintensität **nach der OP** an, wobei 0= kein Schmerz, 10= unerträglicher Schmerz

□ 0 □ 1 □ 2 □ 3 □ 4 □ 5 □ 6 □ 7 □ 8 □ 9 □ 10 keine Schmerzen 🡪 unerträglich

|  | **Gar nicht zutreffend** | **Etwas zutreffend** | **Ziemlich zutreffend** | **Sehr stark zutreffend** |
| --- | --- | --- | --- | --- |
| **12. Hatten Sie weniger Energie als üblich?** |  |  |  |  |
| **13. Waren Sie bei Ihrer Arbeit oder anderen tagtäglichen Beschäftigungen eingeschränkt?** |  |  |  |  |
| **14. Waren Sie bei Ihren Hobbys oder anderen Freizeitaktivitäten eingeschränkt?** |  |  |  |  |
| **15. Mussten Sie sich häufiger als vor der OP ausruhen?** |  |  |  |  |
| **16. Waren Sie häufiger müde als vor der OP?** |  |  |  |  |
| **17. Hatten Sie Probleme dabei, sich wie üblich mit Freunden oder der Familie zu treffen?** |  |  |  |  |
| **18. Hatten Sie Probleme damit, mit Freunden oder Ihrer Familie über Ihre krankheitsbedingten Gefühle zu sprechen?** |  |  |  |  |
| **19. Fühlten Sie sich gestresster als vor der OP?** |  |  |  |  |
| **20. Machten Sie sich Gedanken über Ihre Gesundheit in der Zukunft?** |  |  |  |  |
| **21. Machten Sie sich Sorgen über Ihre Familie in der Zukunft aufgrund Ihrer Erkrankung?** |  |  |  |  |
| **22. Hat Ihr körperlicher Zustand oder Ihre medizinische Behandlung Sie in finanzielle Schwierigkeiten gebracht?** |  |  |  |  |
| **23. Hatten Sie Schwierigkeiten, sich auf etwas zu konzentrieren, z.B. auf die Zeitung oder den Fernseher?** |  |  |  |  |
| **24. Fühlten Sie sich angespannter als vor der OP?** |  |  |  |  |
| **25. Fühlten Sie sich niedergeschlagener als vor der OP?** |  |  |  |  |
| **26. Waren Sie trotz ihrer Erkrankung glücklich?** |  |  |  |  |

Im Folgenden sind Tätigkeiten beschrieben, die Sie eventuell an einem normalen Tag ausüben. Waren sie durch Ihren Gesundheitszustand **nach** der OP bei diesen Tätigkeiten eingeschränkt? Wenn ja, wie stark?

|  | **Gar nicht einge- schränkt** | **Etwas einge- schränkt** | **Ziemlich einge- schränkt** | **Sehr einge- schränkt** |
| --- | --- | --- | --- | --- |
| **27. Anstrengende Tätigkeiten, z.B. Schnell laufen, schwere Gegenstände heben, anstrengenden Sport treiben** |  |  |  |  |
| **28. Mittelschwere Tätigkeiten z.B. einen Tisch verschieben, staubsaugen, Golf spielen** |  |  |  |  |
| **29. Einkaufstaschen tragen oder heben** |  |  |  |  |
| **30. Mehrere Treppenabsätze steigen** |  |  |  |  |
| **31. Einen Treppenabsatz steigen** |  |  |  |  |
| **32. Sich beugen, knien, bücken** |  |  |  |  |
| **33. Mehr als 1 Kilometer zu Fuß gehen** |  |  |  |  |
| **34. Über 500m zu Fuß gehen** |  |  |  |  |
| **35. Weniger als 500m zu Fuß gehen** |  |  |  |  |
| **36. Sich baden oder anziehen** |  |  |  |  |

37. Wie würden Sie Ihren Gesundheitszustand **nach** der OP einschätzen , wenn Sie Schulnoten vergeben?

□ 1 □ 2 □ 3 □ 4 □ 5 □ 6

ausgezeichnet 🡪 Sehr schlecht

38. Wie würden Sie insgesamt Ihre Lebensqualität **nach** der OP einschätzen, wenn Sie Schulnoten vergeben würden?

□ 1 □ 2 □ 3 □ 4 □ 5 □ 6

ausgezeichnet 🡪 Sehr schlecht

39. Wir interessieren uns zudem, wie zufrieden Sie mit der Operation waren. Bitte kreuzen Sie erneut die entsprechende Zahl an!

□ 1 □ 2 □ 3 □ 4 □ 5 □ 6

ausgezeichnet 🡪 Sehr schlecht

40. Geben Sie nun bitte an, wie sich der **Schmerz im Verlauf nach der Operation** entwickelt hat! Kreuzen Sie bitte das entsprechende Feld an.

| Schmerzstärke | **Kein Schmerz** | **Schwacher Schmerz** | **Mittlerer Schmerz** | **Starker Schmerz** | **Sehr starker Schmerz** |
| --- | --- | --- | --- | --- | --- |
| **Direkt nach** der Operation |  |  |  |  |  |
| **1 Monat nach der Operation** |  |  |  |  |  |
| **6 Monate nach der Operation** |  |  |  |  |  |
| **1 Jahr nach der Operation** |  |  |  |  |  |

**Hier ist Platz für Bemerkungen Ihrerseits:**

**Im Vierten Teil des Fragebogens möchten wir Sie gerne zu ihrem aktuellen Befinden befragen.**

1. Bitte geben Sie wieder die für Sie aktuell zutreffende Schmerzintensität an, wobei 0= kein Schmerz, 10= unerträglicher Schmerz bedeutet.

□ 0 □ 1 □ 2 □ 3 □ 4 □ 5 □ 6 □ 7 □ 8 □ 9 □ 10 keine Schmerzen 🡪 unerträglich

2. Wie würden Sie Ihren Gesundheitszustand **aktuell** einschätzen? Verwenden Sie bitte erneut die Schulnoten!

□ 1 □ 2 □ 3 □ 4 □ 5 □ 6

ausgezeichnet 🡪 Sehr schlecht

3. Wie würden Sie insgesamt Ihre Lebensqualität **aktuell** einschätzen? Verwenden Sie bitte erneut die Schulnoten!

□ 1 □ 2 □ 3 □ 4 □ 5 □ 6

ausgezeichnet 🡪 Sehr schlecht

4. Zum Schluss interessiert uns noch, inwieweit jede der folgenden Aussagen auf Sie zutrifft. Bitte kreuzen Sie die zutreffenden Aussagen an!

|  | **Trifft ganz zu** | **Trifft weitgehend zu** | **Trifft weitgehend nicht zu** | **Trifft überhaupt nicht zu** |
| --- | --- | --- | --- | --- |
| **Ich scheine leichter als andere krank zu werden.** |  |  |  |  |
| **Ich bin genauso gesund wie alle anderen, die ich kenne.** |  |  |  |  |
| **Ich erwarte, dass meine Gesundheit nachlässt.** |  |  |  |  |
| **Ich erfreue mich ausgezeichneter Gesundheit.** |  |  |  |  |
